# ﻿Three new species of *Otacilia* Thorell, 1897 (Araneae, Phrurolithidae) from South China

**DOI:** 10.3897/zookeys.1180.108823

**Published:** 2023-09-19

**Authors:** Ming-kang Liu, Zi-min Jiang, Yong-hong Xiao, Ke-ke Liu, Xiang Xu

**Affiliations:** 1 College of Life Science, Jinggangshan University, Ji’an 343009, Jiangxi, China Jinggangshan University Ji’an China; 2 College of Life Science, Hunan Normal University, Changsha 410081, Hunan, China Hunan Normal University Changsha China

**Keywords:** Jiangxi Province, phrurolithid species, spider fauna, taxonomy

## Abstract

Three new *Otacilia* species were collected from Jiangxi Provinces, China during a survey of the spider fauna of the region: *Otaciliaanfu* Liu, **sp. nov.** (♂♀), *O.guanshan* Liu, **sp. nov.** (♂♀), and *O.mingyueshan* Liu, **sp. nov.** (♂♀). All species are described and illustrated with photographs and SEM micrographs, and their distribution is also mapped.

## ﻿Introduction

Over the past four years, the number of phrurolithid species worldwide has increased greatly, especially from China, where the fauna has multiplied several times. Currently, there are 366 species belong to 24 genera worldwide (WSC 2023). Almost half of them (173 species) from 14 genera were recorded in China. The total number is still increasing, based on several arachnologists’ work, such as Dr Feng Zhang, Dr Chi Jin, Dr Xiang Xu, Dr Shu-qiang Li, and Dr Ke-ke Liu, who reported nearly 100 new species in the past five years (Liu et al. 2019, 2020a, b, 2021, 2022a, b, 2023; Yao et al. 2019a, b; Liang et al. 2021; Mu and Zhang 2021, 2022; Jin et al. 2022; Mu et al. 2022a, b). All these species were discovered in southern China, and many new genera were erected at the same time. However, there are still many unknown phrurolithid species from southern China with unusual morphological characteristics.

The genus *Otacilia* Thorell, 1897 is the largest genus among the genera of Phrurolithidae (WSC 2023). It was intertwined with *Phrurolithus* C.L. Koch, 1839 for a long time. Zamani and Marusik (2020) made the distinction between *Otacilia* and *Phrurolithus* based on many morphological characters and transferred 27 species of *Phrurolithus* to *Otacilia*. Subsequently, the genus *Otacilia* is so large and the morphological variation within its supposed members so broad that the assignment of some species to *Otacilia* has been questioned. Since then, many species have been newly combined or re-assigned in new genera, such as *Aculithus* Liu & Li, 2022, *Alboculus* Liu, 2020, *Corealithus* Kamura, 2021, *Grandilithus* Liu & Li, 2022, *Lunalithus* Kamura, 2022, *Pennalithus* Kamura, 2021, and *Xilithus* Liu & Li, 2022 (Liu et al. 2020a, 2022a; Kamura 2021, 2022). However, there still remain many species misplaced in this genus.

During the past 10 years, we have focused on sac spiders in Jiangxi Province, southern China, and 62 phrurolithid species with unusual characters have been found (Liu et al. 2020a, 2020b, 2021, 2022a). There are still many unknown phrurolithidae species from this province with unusual morphological characteristics. In the last year, we examined the phrurolithid specimens collected from Jiangxi Province, and three new *Otacilia* species were discovered. The goal of this paper is to describe these new species.

## ﻿Material and method

Specimens were examined using a SZ6100 stereomicroscope. Both male and female genital organs were dissected and examined in 80% ethanol using an Olympus CX43 compound microscope with a KUY NICE CCD camera. The epigynes were cleared with pancreatin solution. Specimens, including dissected male palps and epigynes, were preserved in 75% ethanol after examination. For SEM photographs, the specimens were kept under natural dry conditions, coated with gold with a small ion-sputtering apparatus ETD-2000, and photographed with a Zeiss EVO LS15 scanning electron microscope. All other specimens are deposited in the Animal Specimen Museum, College of Life Science, Jinggangshan University (**ASM-JGSU**).

The measurements were taken using a stereomicroscope (Axio Vision SE64 rel. 4.8.3) and are given in millimetres. The body lengths of all specimens exclude the chelicerae and spinnerets. Terminology of the male and female genitalia follows Liu et al. (2022a, b, 2023).

Leg measurements are given as total length (including femur, patella, tibia, metatarsus, tarsus). Leg spines are documented by dividing each leg segment into four aspects: dorsal (d), prolateral (p), and retrolateral (r), and indicating the ventral (v) spines as single (1) or paired (2), e.g., femur I d2, p1111; tibia d1, I v2222. The abbreviations used in the figures and text are as follows:

Eyes

**ALE** anterior lateral eye;

**AME** anterior median eye;

**MOA** median ocular area;

**PLE** posterior lateral eye;

**PME** posterior median eye.

Male palp

**dTA** distal tegular apophysis;

**EBP** embolic basal process;

**Em** embolus;

**Gr** groove;

**FA** femoral apophysis;

**PTA** prolateral tibial apophysis;

**rTA** retrolateral tegular apophysis;

**RTA** retrolateral tibial apophysis;

**SD** sperm duct;

**VTA** ventro-retrolateral tibial apophysis.

Epigyne

**Bu** bursa;

**CD** copulatory duct;

**CO** copulatory opening;

**CT** connecting tube;

**FD** fertilisation duct;

**GA** glandular appendage;

**MS** median septum;

**Spe** spermathecae.

## ﻿Taxonomy

### ﻿Family Phrurolithidae Banks, 1892

**Genus *Otacilia* Thorell, 1897**

#### ﻿ Otacilia anfu

Taxon classificationAnimaliaAraneaePhrurolithidae

Liu sp. nov.

35C7B220-E374-544C-B122-2DEE5C588BA5

https://zoobank.org/3DB14EA7-34DF-4D89-A932-543EFA2534F6

**Figure 1.**
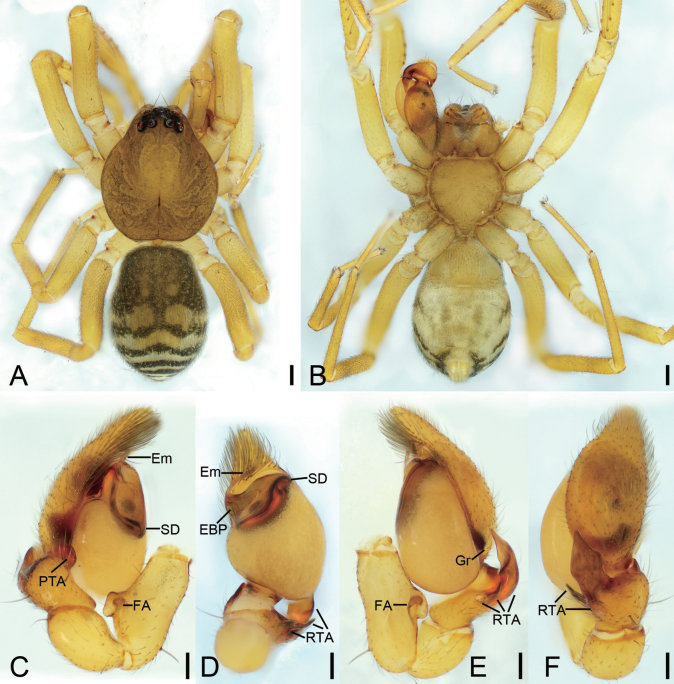
*Otaciliaanfu* sp. nov., male holotype **A** habitus, dorsal view **B** same, ventral view **C** palp, prolateral view **D** same, ventral view **E** same, retrolateral view **F** same, dorso-retrolateral view. Abbreviations: EBP – embolic basal process, Em – embolus, FA – femoral apophysis, Gr – groove, PTA – prolateral tibial apophysis, RTA – retrolateral tibial apophysis, SD – sperm duct. Scale bars: 0.2 mm (**A, B**); 0.1 mm (**C–F**).

**Figure 2.**
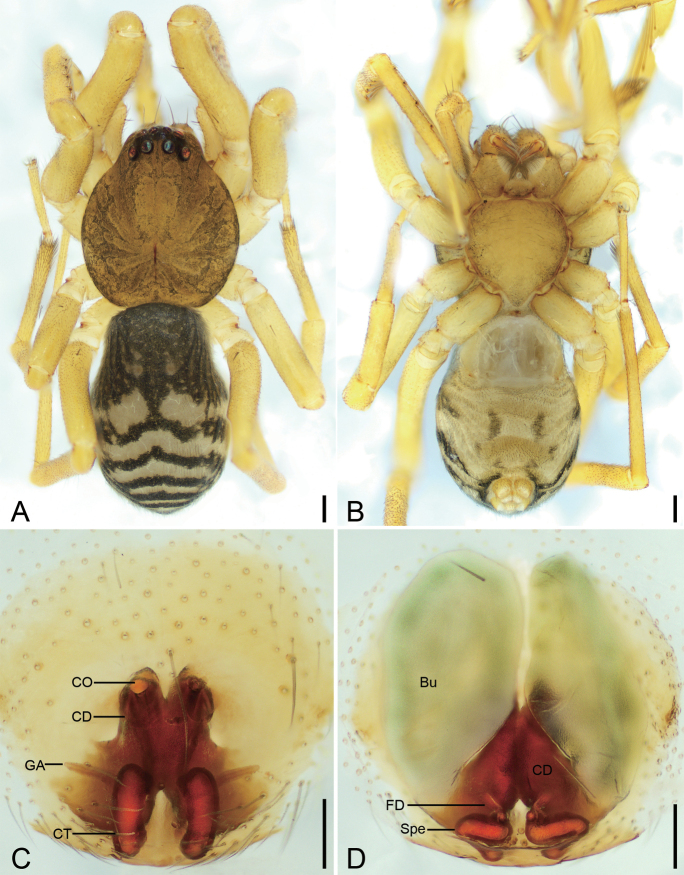
*Otaciliaanfu* sp. nov., female paratype **A** habitus, dorsal view **B** same, ventral view **C** epigyne, ventral view **D** vulva, dorsal view. Abbreviations: Bu – bursa, CD – copulatory duct, CO – copulatory opening, CT – connecting tube, FD – fertilisation ducts, GA – glandular appendage, Spe – spermathecae. Scale bars: 0.2 mm (**A, B**); 0.1 mm (**C, D**).

Figs 1 , 2 , 7A , 8A

##### Type materials.

***Holotype***: ♂ (Phu-142), China, Jiangxi Province, Ji’an City, Anfu County, Yangshimu Scenic Spot, Yunzhong Slide Rail, 27°33'04.43"N, 114°15'00.52"E, 1558 m, 3 January 2022, leg. Ke-ke Liu et al. ***Paratypes***: 2 ♂, 1 ♀, same data as holotype (Phu-142).

##### Etymology.

The specific name derived from Anfu County, where the type locality is situated; it is treated as a noun in apposition.

##### Diagnosis.

The male of the new species is similar to *Otaciliaarcuata* Mu & Zhang, 2021 in having a large, finger-shaped retrolateral apophysis and swollen tegulum (Mu and Zhang 2021: 543, fig. 6F), but it can be separated from it by the carapace lacking broad dark brown bands around submargin (vs present) (cf. Fig. 1A, B and Mu and Zhang 2021: 543, fig. 6A), the legs lacking brown annulations (vs present) (cf. Fig. 1A, B and Mu and Zhang 2021: 543, fig. 6A), the sharp, spine-like intermediate retrolateral tibial apophysis (vs small and tooth-like) (cf. Fig. 1D–F and Mu and Zhang 2021: 543, fig. 6E, F), and the long, spine-like embolus (vs shorter) (cf. Fig. 1D–F and Mu and Zhang 2021: 543, fig. 6E, F). The female resemble that of *O.arcuata* in having the spermathecae with an obvious transverse part and slender glandular appendages (Mu and Zhang 2021: 543, fig. 6G, H), but can be separated from it by the relatively long copulatory ducts (vs very short) (cf. Fig. 2C, D and Mu and Zhang 2021: fig. 6G, H) and pair of parallel, sausage-shaped connecting tubes (vs C-shaped) (cf. Fig. 2C and Mu and Zhang 2021: 543, fig. 6G).

##### Description.

**Male** (holotype). ***Habitus*** as in Fig. 1A, B. Total length 2.84, carapace 1.34 long, 1.20 wide. Eye sizes and interdistances: AME 0.07, ALE 0.10, PME 0.09, PLE 0.08; AME–AME 0.04, AME–ALE 0.01, PME–PME 0.10, PME–PLE 0.04, AME–PME 0.07, AME–PLE 0.15, ALE–ALE 0.19, PLE–PLE 0.35, ALE–PLE 0.07. MOA 0.20 long, frontal width 0.16, posterior width 0.27. Chelicerae with three promarginal and six retromarginal teeth. Sternum (Fig. 1B), nearly as long as wide, posterior end blunt. Leg measurements: I 4.43 (1.22, 0.47, 1.26, 1.05, 0.43); II 3.78 (1, 0.38, 0.93, 0.89, 0.58); III 3.22 (0.91, 0.39, 0.67, 0.86, 0.39); IV 4.78 (1.34, 0.43, 1.01, 1.33, 0.67). Leg spination (Fig. 1A, B): femora I d11, p1111, II d1, p11, III d1, IV d1; tibiae I v222222, II v222222, metatarsi I v2222, II v2221. Abdomen (Fig. 1A, B) 1.41 long, 1.01 wide, dorsal scutum covering more than 1/2 length of abdomen.

***Colouration*** (Fig. 1A, B). Carapace yellow-brown, with radial, irregular, dark yellow-brown mottled markings on surface. Chelicerae, endites and labium yellow-brown. Sternum yellow; lateral margins with dark mottled markings. Legs yellow, without brown annulations. Abdomen brown, with pair of small round and pair of large oval yellow spots on medial dorsal scutum, three pale chevron-shaped stripes on subposterior part, and two yellowish arc-shaped stripes posteriorly; venter with blurred H-shaped and pair of sloping markings posteriorly.

***Palp*** (Figs 1C−F, 7A). Femoral apophysis (FA) well developed, as wide as half of femoral length. Tibia with four apophyses: a n-shaped prolateral apophysis (PTA), relatively broad, with extruding margin; three retrolateral apophyses (RTA): a tooth-like retrolateral apophysis in ventral view, shorter than other two retrolateral apophyses; a small hook-shaped retrolateral apophysis, longer than previous one, but shorter than last one; a large finger-like, thick retrolateral apophysis in retrolateral view, conical in dorsal view, as long as tibial length, medially thickened, apex blunt, bent inwards toward the small cymbial groove (Gr). Bulb broadly oval, with V-shaped sperm duct (SD) in ventral view, apophyses absent. Embolus (Em) spine-like, with a lamellar embolic basal process (EBP).

**Female. *Habitus*** as in Fig. 2A, B. Total length 2.97, carapace 1.37 long, 1.21 wide. Eye sizes and interdistances: AME 0.07, ALE 0.09, PME 0.08, PLE 0.09, AME–AME 0.03, AME–ALE 0.01, PME–PME 0.09, PME–PLE 0.05, AME–PME 0.08, AME–PLE 0.15, ALE–ALE 0.19, PLE–PLE 0.36, ALE–PLE 0.07. MOA 0.21 long, frontal width 0.17, posterior width 0.26. Leg measurements: I 4.77 (1.26, 0.51, 1.35, 1.14, 0.51); II 4.03 (1.06, 0.46, 1.01, 0.93, 0.57); III 3.52 (0.93, 0.41, 0.7, 0.93, 0.55); IV 4.94 (1.33, 0.47, 1.12, 1.31, 0.71). Leg spination (Fig. 2A, B): femora femora I d11 p1111, II d1 p11, III d1, IV d1; tibiae I v2222222, II v222222, metatarsi I v2222, II v2222. Pedicel 0.03 long. Abdomen (Fig. 2A, B) 1.65 long, 1.07 wide.

***Colouration*** (Fig. 2A, B). Paler than male.

***Epigyne*** (Fig. 2C, D). Epigynal plate W-shaped through integument, median septum absent. Copulatory openings (CO) anteromedially located, slightly separated. Copulatory ducts (CD) broad, touching, posteriorly with pair of soybean-shaped, transparent bursae laterally. Bursae (Bu) large, bean-shaped, slightly separated, nearly covering 4/5 of epigynal plate. Glandular appendages (GA) very long, located on posterior of copulatory ducts. Connecting tubes (CT) sausage-shaped, separated by half of their length. Spermathecae (Spe) S-shaped, transverse, slightly separated. Fertilisation ducts (FD) short, located at anterior of spermathecae, directed anterolaterally.

##### Distribution.

Known only from the type locality in Jiangxi Province, China (Fig. 9).

#### ﻿ Otacilia guanshan

Taxon classificationAnimaliaAraneaePhrurolithidae

Liu sp. nov.

3E708E4A-61A3-57EC-939B-737BDA89089C

https://zoobank.org/703BAFC2-77B6-4CF9-88D3-8F30E449E19C

**Figure 3.**
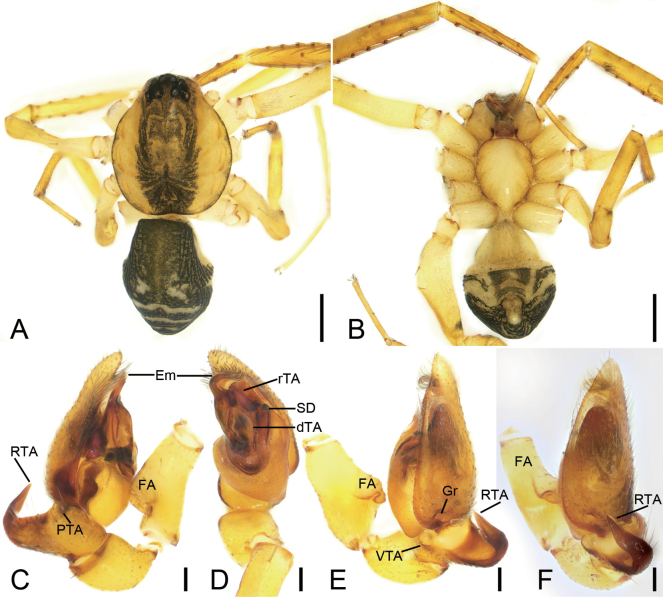
*Otaciliaguanshan* sp. nov., male holotype **A** habitus, dorsal view **B** same, ventral view **C** palp, prolateral view **D** same, ventral view **E** same, retrolateral view **F** same, dorso-retrolateral view. Abbreviations: dTA – distal tegular apophysis, Em – embolus, FA – femoral apophysis, Gr – groove, PTA – prolateral tibial apophysis, rTA – retrolateral tegular apophysis, RTA – retrolateral tibial apophysis, SD – sperm duct, VTA – ventro-retrolateral tibial apophysis. Scale bars: 0.5 mm (**A, B**); 0.1 mm (**C–F**).

**Figure 4.**
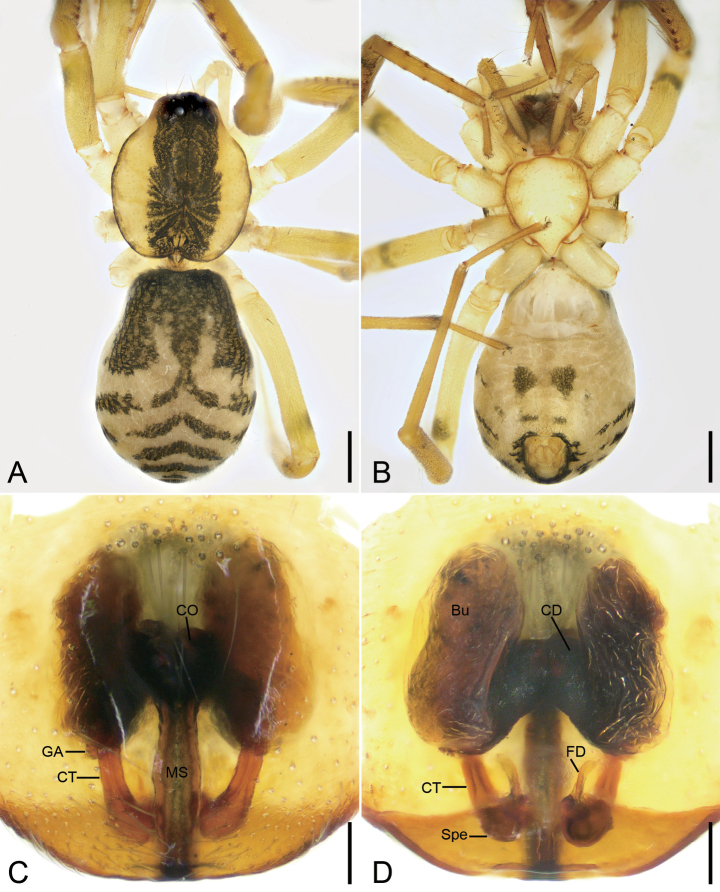
*Otaciliaguanshan* sp. nov., female paratype **A** habitus, dorsal view **B** same, ventral view **C** epigyne, ventral view **D** vulva, dorsal view. Abbreviations: Bu – bursa, CD – copulatory duct, CO – copulatory opening, CT – connecting tube, FD – fertilisation ducts, GA – glandular appendage, MS – median septum, Spe – spermathecae. Scale bars: 0.5 mm (**A, B**); 0.1 mm (**C, D**).

Figs 3 , 4 , 7B , 8B, C

##### Type materials.

***Holotype***: ♂ (Phu-158), China, Jiangxi Province, Yichun City, Yifeng County, Guanshan National Nature Reserve, 28°32'22.88"N, 114°35'47.00"E, 719 m, 25 February 2023, leg. Ke-ke Liu et al. ***Paratypes***: 1 ♂, 4 ♀, with the same data as holotype (Phu-158); 1 ♀, Donghe Station, 28°33'16.15"N, 114°34'53.72"E, 439 m, other data as previous (Phu-158).

##### Etymology.

The specific name derived from the type locality, Guanshan National Nature Reserve; used as a noun in apposition.

##### Diagnosis.

**Figure 5.**
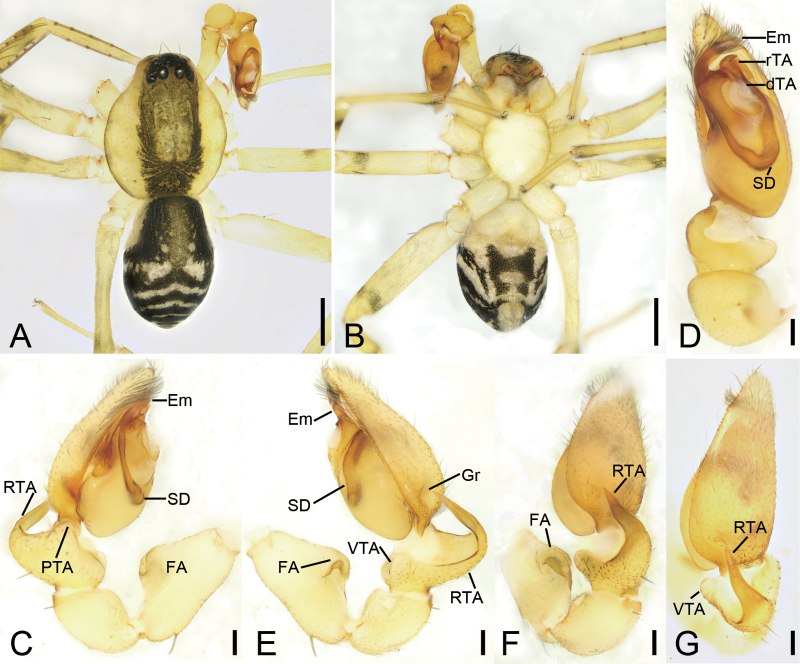
*Otaciliamingyueshan* sp. nov., male holotype **A** habitus, dorsal view **B** same, ventral view **C** palp, prolateral view **D** same, ventral view **E** same, retrolateral view **F** same, dorso-retrolateral view. Abbreviations: dTA – distal tegular apophysis, Em – embolus, FA – femoral apophysis, Gr – groove, PTA – prolateral tibial apophysis, rTA – retrolateral tegular apophysis, RTA – retrolateral tibial apophysis, SD – sperm duct, VTA – ventro-retrolateral tibial apophysis. Scale bars: 0.5 mm (**A, B**); 0.1 mm (**C–F**).

**Figure 6.**
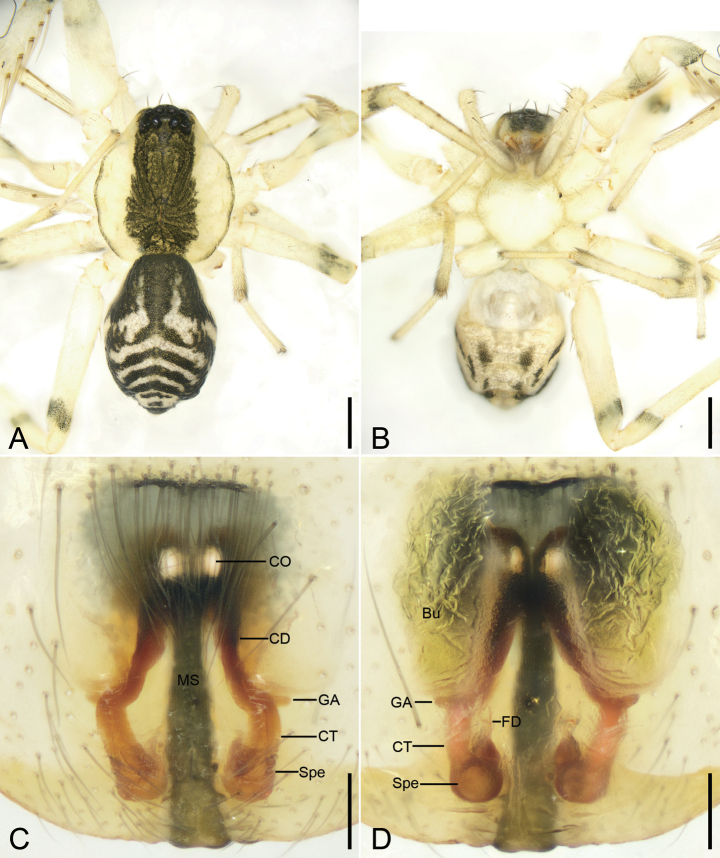
*Otaciliamingyueshan* sp. nov., female paratype **A** habitus, dorsal view **B** same, ventral view **C** epigyne, ventral view **D** vulva, dorsal view. Abbreviations: Bu – bursa, CD – copulatory duct, CO – copulatory opening, CT – connecting tube, FD – fertilisation ducts, GA – glandular appendage, MS – median septum, Spe – spermathecae. Scale bars: 0.5 mm (**A, B**); 0.1 mm (**C, D**).

The male of the new species is similar to *O.mingyueshan* sp. nov. and *O.wugongshanica* Liu, 2020 (Liu et al. 2020b: 17, fig. 12A, D, E) in having the carapace with broad, dark-brown, mottled markings medially and a clavate retrolateral tegular apophysis, but it can be separated from them by the retrolateral tibial apophysis with thicker apex (vs moderate in *O.mingyueshan* sp. nov.; thin in *O.wugongshanica*) (cf. Fig. 3E, F and Fig. 5E, F and Liu et al. 2020b: 17, fig. 12E, F) and the hook-shaped embolus strongly curved backward (vs crescent-shaped embolus curved forward in *O.mingyueshan* sp. nov. and in *O.wugongshanica*) (cf. Fig. 3D and Fig. 5D and Liu et al. 2020b: 17, fig. 12D). The female resemble those of *O.mingyueshan* sp. nov., and *O.wugongshanica* Liu, 2020 (Liu et al. 2020b: 17, fig. 14C, D) in having the bow and arrow-shaped mark on the epigynal plate, but the new species can be separated from them by having the fovea without a distinct sclerotised transverse anterior margin (vs with a short, sclerotised transverse margin in *O.mingyueshan* sp. nov. and *O.wugongshanica* Liu, 2020) (cf. Fig. 4C and Fig. 5C and Liu et al. 2020b: 17, fig. 14C), the spindle shaped median septum (vs trapezoidal in *O.mingyueshan* sp. nov.; same in *O.wugongshanica*) (cf. Fig. 4C and Fig. 6C and Liu et al. 2020b: 17, fig. 14C), the straightly sloping connecting tubes (vs same in *O.mingyueshan* sp. nov.; curved in *O.wugongshanica*) (cf. Fig. 4D and Fig. 6D and Liu et al. 2020b: 17, fig. 14D); and the relatively widely separated spermathecae (vs widely separated in *O.mingyueshan* sp. nov.; touching in *O.wugongshanica*) (cf. Fig. 4D and Fig. 6D and Liu et al. 2020b: 17, fig. 14D).

##### Description.

**Male** (holotype). ***Habitus*** as in Fig. 3A, B. Total length 2.93, carapace 1.50 long, 1.28 wide. Eye sizes and interdistances: AME 0.07, ALE 0.09, PME 0.07, PLE 0.08; AME–AME 0.07, AME–ALE 0.03, PME–PME 0.13, PME–PLE 0.07, AME–PME 0.09, AME–PLE 0.19, ALE–ALE 0.24, PLE–PLE 0.39, ALE–PLE 0.09. MOA 0.24 long, frontal width 0.19, posterior width 0.26. Chelicerae with three promarginal and five retromarginal teeth. Sternum (Fig. 3B) with shallow groove along lateral margins, posterior end triangular, relatively blunt. Leg measurements: I 4.21 (1.23, 0.44, 1.18, 0.81, 0.55); II 4.11 (1.2, 0.36, 1.19, 0.73, 0.63); III 3.75 (1.05, 0.43, 0.88, 0.77, 0.62); IV 6.02 (1.26, 0.38, 1.83, 1.63, 0.92). Leg spination (Fig. 3A, B): femora I d1, p1111, II d1, p111, III d1, IV d1; tibiae I v2222222, II v2222222, metatarsi I v2222, II v2222. Pedicel 0.12 long. Abdomen (Fig. 3A, B) 1.83 long, 0.97 wide, weak dorsal scutum in anterior half.

***Colouration*** (Fig. 3A, B). Carapace yellow with conspicuous, irregular, dark-yellow-brown mottled markings radially along midline and arc-shaped dark stripes around margin. Chelicerae and endites yellow-brown. Labium yellow-brown. Sternum yellow. Legs yellow. Abdomen black-brown, with pair of small, oval and pair of irregular yellowish spots on posterior dorsal scutum, three pale chevron-shaped stripes on subposterior part, and indistinct, yellowish, arc-shaped stripe posteriorly; venter with trapezoidal marking and pair of sloping stripes posteriorly.

**Figure 7.**
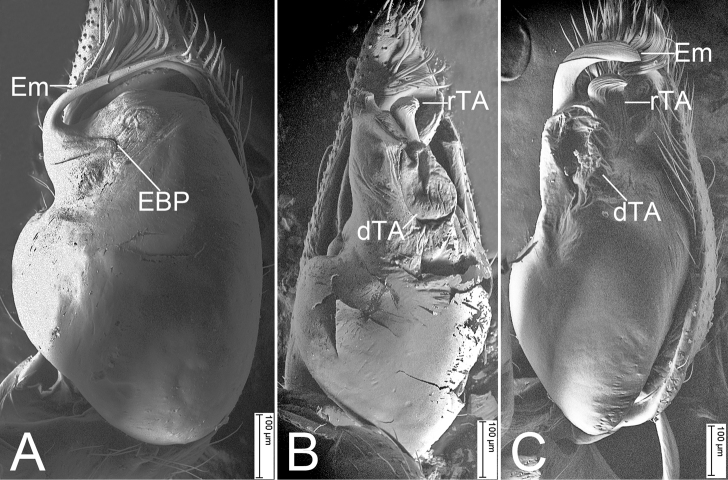
SEM micrographs of *Otacilia* spp. from China, palp of male paratype **A***O.anfu* sp. nov., ventral view **B***O.guanshan* sp. nov., ventral view **C***O.mingyueshan* sp. nov., ventral view. Abbreviations: dTA – distal tegular apophysis, EBP – embolic basal process, Em – embolus, rTA – distal tegular apophysis.

***Palp*** (Figs 3C−F, 7B). Femoral apophysis (FA) well developed, as wide as half of femoral width. Tibia with three apophyses: a small, tubercle-like ventro-retrolateral apophysis (VTA) proximally, a large, thick, very strong retrolateral apophysis (RTA), longer than tibia, strongly bent inwards toward concaved cymbial groove (Gr), and a thickened, ridge-like prolateral apophysis (PTA). Sperm duct (SD) V-shaped in ventral view, reaching subposterior part of tegulum. Retrolateral tegular apophysis (rTA) clavate, thick, directed anterolaterally, shorter than embolus in ventral view. Distal tegular apophysis (dTA) oval, membranous, arising from base of embolus and retrolateral part of sperm duct. Embolus (Em) hook-shaped, strongly curved backward, with a broad base.

**Female. *Habitus*** as in Fig. 4A, B. Total length 3.86, carapace 1.50 long, 1.38 wide. Eye sizes and interdistances: AME 0.08, ALE 0.07, PME 0.08, PLE 0.07, AME–AME 0.04, AME–ALE 0.03, PME–PME 0.12, PME–PLE 0.09, AME–PME 0.11, AME–PLE 0.21, ALE–ALE 0.23, PLE–PLE 0.38, ALE–PLE 0.13. MOA 0.25 long, frontal width 0.19, posterior width 0.26. Chelicerae with three promarginal and seven retromarginal teeth. Leg measurements: I 5.99 (1.53, 0.65, 1.64, 1.64, 0.53); II 4.47 (1.27, 0.5, 0.96, 1.23, 0.51); III 4.22 (0.94, 0.51, 0.93, 1.22, 0.62); IV 6.57 (1.64, 0.65, 1.56, 1.87, 0.85). Leg spination (Fig. 6A, B): femora I d1, p111, II d1, p111, III d1, IV d1; tibiae I v22222222, II v2222222, metatarsi I v2222, II v1222. Pedicel 0.11 long. Abdomen (Fig. 4A, B) 2.20 long, 1.62 wide.

***Colouration*** (Fig. 4A, B). Paler than male. Abdomen yellow to black-brown, dorsally with four dark-brown, chevron-shaped stripes on submedial part, and two arc-shaped, short stripes posteriorly; venter with pair of triangular markings and pair of transverse stripes posteriorly.

***Epigyne*** (Fig. 4C, D). Epigynal plate with bow and arrow-shaped mark, posteriorly with elongated subspindle-shaped median septum (MS). Fovea located anteriorly, without indistinct sclerotised transverse anterior margin. Copulatory openings (CO) oval, arising from anterior part of median septum. Copulatory ducts (CD) strongly sclerotised, relatively broad, splayed, longer than connecting tubes. Bursae (Bu) large, bean-shaped, widely separated, nearly covering 1/2 of epigynal plate. Glandular appendages (GA) small, indistinct in ventral view. Connecting tubes (CT) short, slightly convergent, widely separated. Spermathecae (Spe) small, globular, separated by their length, located on subposterior part of endogyne. Fertilisation ducts (FD) short, directed anteriorly.

##### Distribution.

Known only from the type locality in Jiangxi Province, China (Fig. 9).

#### ﻿ Otacilia mingyueshan

Taxon classificationAnimaliaAraneaePhrurolithidae

Liu sp. nov.

06407DC2-4203-5CD2-988E-74E979998095

https://zoobank.org/EA43D15D-4ED7-4BBD-B556-595A651A8385

**Figure 8.**
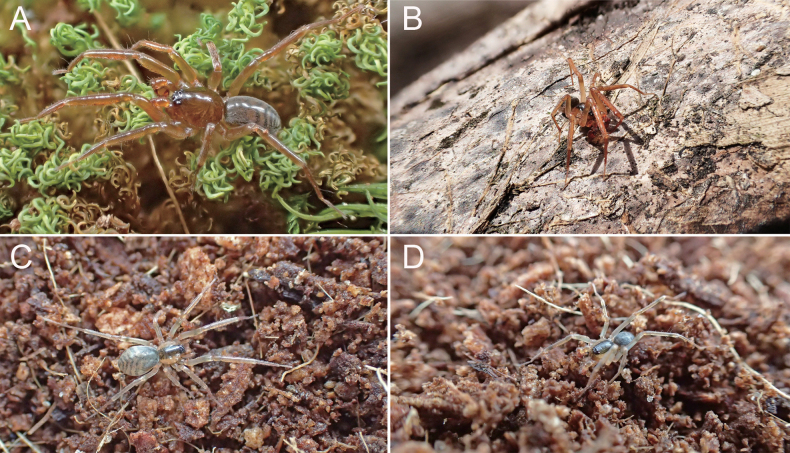
Photographs of living *Otacilia* specimens from Jiangxi Province, China **A***O.anfu* sp. nov. **B, C***O.guanshan* sp. nov. **D***O.mingyueshan* sp. nov.

Figs 5 , 6 , 7C , 8D

##### Type materials.

***Holotype***: ♂ (Phu-151), China, Jiangxi Province, Yichun City, Yuanzhou District, Wenquan Town, Mingyueshan National Forest Park, near Yungu Waterfall, 27°35'39.35"N, 114°16'10.04"E, 711 m, 15 October 2022, leg. Ke-ke Liu et al. ***Paratypes***: 2 ♂, 3 ♀, with the same data as holotype (Phu-151).

##### Etymology.

The specific name derived from the type locality, Mingyueshan National Forest Park; noun in apposition.

##### Diagnosis.

The male of the new species is similar to *O.guanshan* sp. nov. and *O.wugongshanica* Liu, 2020 (Liu et al. 2020b: 17, fig. 12A, D, E) in having the carapace with broad, dark-brown, mottled markings medially and a clavate retrolateral tegular apophysis, but it can be separated from them by the retrolateral tibial apophysis with moderate apex (vs thicker in *O.guanshan* sp. nov. and thin in *O.wugongshanica*) (cf. Fig. 5F, G and Fig. 3E, F and Liu et al. 2020b: 17, fig. 12E, F), the reniform distal tegular apophysis (vs oval in *O.guanshan* sp. nov. and similar in *O.wugongshanica*) (cf. Fig. 5D and Fig. 3D and Liu et al. 2020b: 17, fig. 12D, E), and the crescent-shaped embolus (vs similar in *O.wugongshanica* and hook-shaped in *O.guanshan* sp. nov.) (cf. Fig. 5D, Fig. 3D, and Liu et al. 2020b: 17, fig. 12D). The female resembles those of *O.guanshan* sp. nov., and *O.wugongshanica* Liu, 2020 (Liu et al. 2020b: 17, fig. 14C, D) in having the bow and arrow-shaped mark in epigynal plate, but the new species can be separated from them by the trapezoidal median septum (vs spindle-shaped in *O.guanshan* sp. nov. and *O.wugongshanica*) (cf. Fig. 6C, Fig. 4C, and Liu et al. 2020b: 17, fig. 14C), the straightly sloping connecting tubes (vs similar in *O.guanshan* sp. nov. and curved in *O.wugongshanica*) (cf. Fig. 6D, Fig. 4D, and Liu et al. 2020b: 17, fig. 14D) and the widely separated spermathecae (vs relatively widely separated in *O.guanshan* sp. nov. and touching in *O.wugongshanica*) (cf. Fig. 6D and Fig. 4D and Liu et al. 2020b: 17, fig. 14D).

##### Description.

**Male** (holotype). ***Habitus*** as in Fig. 5A, B. Total length 3.02, carapace 1.50 long, 1.26 wide. Eye sizes and interdistances: AME 0.06, ALE 0.08, PME 0.07, PLE 0.08; AME–AME 0.06, AME–ALE 0.03, PME–PME 0.12, PME–PLE 0.05, AME–PME 0.11, AME–PLE 0.18, ALE–ALE 0.24, PLE–PLE 0.39, ALE–PLE 0.09. MOA 0.24 long, frontal width 0.20, posterior width 0.28. Chelicerae with three promarginal and six retromarginal teeth. Sternum (Fig. 5B), posterior end triangular, relatively blunt. Leg measurements: I 5 (1.34, 0.43, 1.43, 1.12, 0.68); II 4.73 (1.2, 0.43, 1.26, 1.13, 0.71); III 4.12 (1.07, 0.32, 0.93, 1.14, 0.66); IV 6.16 (1.65, 0.46, 1.24, 1.87, 0.94). Leg spination (Fig. 5A, B): femora I d1, p1111, II d1, p11, III d1, IV d1; tibiae I v222222, II v222222, metatarsi I v2222, II v2222. Pedicel 0.09 long. Abdomen (Fig. 5A, B) 1.56 long, 0.97 wide, weak dorsal scutum in anterior half.

***Colouration*** (Fig. 5A, B). Carapace yellow with conspicuous, irregular, dark-yellow-brown mottled markings radially along midline and arc-shaped dark stripes around margin. Chelicerae yellow-brown. Endites and labium yellow. Sternum yellowish. Legs yellow, with conspicuous annulation on femora. Abdomen black-brown, with pair of small, oval and pair of large, irregular yellowish spots on posterior dorsal scutum, three pale chevron-shaped stripes on submedial part, and indistinct yellowish arc-shaped stripe posteriorly; venter with H-shaped and pair of sloping markings posteriorly.

***Palp*** (Figs 5C−G, 7C). Femoral apophysis (FA) well developed, nearly as wide as half of femoral width. Tibia with three apophyses: one small, tubercle-like ventro-retrolateral apophysis (VTA) proximally; one large retrolateral apophysis (RTA), longer than tibia, tapering from broad base to spine-like apex in dorsal view, bent inwards toward cymbial groove (Gr); one thickened, ridge-like prolateral apophysis (PTA). Sperm duct (SD) V-shaped in ventral view, reaching subposterior part of tegulum. Retrolateral tegular apophysis (rTA) fan-shaped, thick, directed anterolaterally, shorter than embolus and distal tegular apophysis in ventral view. Distal tegular apophysis (dTA) reniform, membranous, arising from base of embolus and retrolateral part of sperm duct. Embolus (Em) crescent-shaped, strongly curved, relatively thick, with a broad base.

**Female. *Habitus*** as in Fig. 6A, B. Total length 3.08, carapace 1.50 long, 1.37 wide. Eye sizes and interdistances: AME 0.06, ALE 0.07, PME 0.06, PLE 0.07, AME–AME 0.07, AME–ALE 0.04, PME–PME 0.14, PME–PLE 0.08, AME–PME 0.11, AME–PLE 0.19, ALE–ALE 0.29, PLE–PLE 0.40, ALE–PLE 0.11. MOA 0.23 long, frontal width 0.19, posterior width 0.25. Leg measurements: I 6.11 (1.65, 0.61, 1.88, 1.26, 0.71); II 5.22 (1.29, 0.54, 1.47, 1.25, 0.67); III 4.15 (1.05, 0.46, 0.98, 1.07, 0.59); IV 6.03 (1.63, 0.47, 1.53, 1.51, 0.89). Leg spination (Fig. 6A, B): femora I d1, p1111, II d1, p111, III d1, IV d1; tibiae I v2222222, II v222222, metatarsi I v2222, II v222. Pedicel 0.08 long. Abdomen (Fig. 6A, B) 1.59 long, 1.18 wide.

***Colouration*** (Fig. 6A, B). Paler than male.

***Epigyne*** (Fig. 6C, D). Epigynal plate bow- and arrow-shaped, posteriorly with elongated trapezoidal median septum (MS). Fovea located anteriorly, separated by strongly sclerotised transverse margin. Copulatory openings (CO) oval, touching at margin, arising from anterior part of median septum. Copulatory ducts (CD) splayed, longer than connecting tubes. Bursae (Bu) large, bean-shaped, widely separated, nearly covering more than 1/2 of epigynal plate. Glandular appendages (GA) small, directed laterally in ventral view. Connecting tubes (CT) relatively broad, very short, nearly parallel in ventral view. Spermathecae (Spe) globular, separated by more than their length, located on subposterior part of endogyne. Fertilisation ducts (FD) short, located at anterior of spermathecae, directed anteriorly.

##### Distribution.

**Figure 9.**
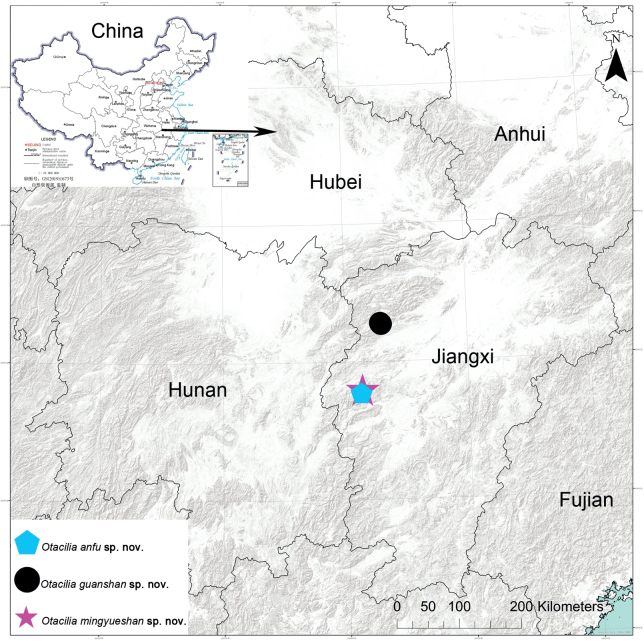
Records of *Otaciliaanfu* sp. nov., *O.guanshan* sp. nov., and *O.mingyueshan* sp. nov. in Jiangxi Province, China.

Known only from the type locality in Jiangxi Province, China (Fig. 9).

## Supplementary Material

XML Treatment for Otacilia anfu

XML Treatment for Otacilia guanshan

XML Treatment for Otacilia mingyueshan

## ﻿Additional information

### Author ORCIDs

Ming-kang Liu https://orcid.org/0009-0005-5607-2323

Zi-min Jiang https://orcid.org/0000-0001-9044-3312

Yong-hong Xiao https://orcid.org/0000-0002-8074-9366

Ke-ke Liu https://orcid.org/0000-0001-7822-3667

Xiang Xu https://orcid.org/0000-0001-9485-5373

### Data availability

All of the data that support the findings of this study are available in the main text.
